# Toll-Like Receptor 9-Mediated Neuronal Innate Immune Reaction Is Associated with Initiating a Pro-Regenerative State in Neurons of the Dorsal Root Ganglia Non-Associated with Sciatic Nerve Lesion

**DOI:** 10.3390/ijms22147446

**Published:** 2021-07-12

**Authors:** Petr Dubový, Ivana Hradilová-Svíženská, Václav Brázda, Marek Joukal

**Affiliations:** Cellular and Molecular Research Group, Department of Anatomy, Faculty of Medicine, Masaryk University, Kamenice 3, CZ-62500 Brno, Czech Republic; isvizen@med.muni.cz (I.H.-S.); vbrazda@med.muni.cz (V.B.); mjoukal@med.muni.cz (M.J.)

**Keywords:** sciatic nerve, compression, transection, mitochondrial DNA, the endoplasmic reticulum, early endosomes, axon regeneration

## Abstract

One of the changes brought about by Wallerian degeneration distal to nerve injury is disintegration of axonal mitochondria and consequent leakage of mitochondrial DNA (mtDNA)—the natural ligand for the toll-like receptor 9 (TLR9). RT-PCR and immunohistochemical or Western blot analyses were used to detect TLR9 mRNA and protein respectively in the lumbar (L4-L5) and cervical (C7-C8) dorsal root ganglia (DRG) ipsilateral and contralateral to a sterile unilateral sciatic nerve compression or transection. The unilateral sciatic nerve lesions led to bilateral increases in levels of both TLR9 mRNA and protein not only in the lumbar but also in the remote cervical DRG compared with naive or sham-operated controls. This upregulation of TLR9 was linked to activation of the Nuclear Factor kappa B (NFκB) and nuclear translocation of the Signal Transducer and Activator of Transcription 3 (STAT3), implying innate neuronal immune reaction and a pro-regenerative state in uninjured primary sensory neurons of the cervical DRG. The relationship of TLR9 to the induction of a pro-regenerative state in the cervical DRG neurons was confirmed by the shorter lengths of regenerated axons distal to ulnar nerve crush following a previous sciatic nerve lesion and intrathecal chloroquine injection compared with control rats. The results suggest that a systemic innate immune reaction not only triggers the regenerative state of axotomized DRG neurons but also induces a pro-regenerative state further along the neural axis after unilateral nerve injury.

## 1. Introduction

The primary sensory neurons of dorsal root ganglia (DRG) sendoff afferent axons, whose peripheral branches extend through peripheral nerves to the target tissue. The central branches run predominantly through the dorsal roots into the spinal cord. DRG neurons are a useful model to study mechanisms regulating the neuronal regeneration program following axotomy. Injury to peripheral axonal branches induces transcription-dependent changes of regeneration-associated genes and proteins, enhancing the regeneration potential of DRG neurons and thus promoting regeneration of the damaged axons [1,2]. This conditioning lesion of peripheral axons also allows regeneration of central axonal branches, but prior injury to these branches is insufficient to activate the same neuronal regeneration program [3,4,5].

Wallerian degeneration (WD) is a cascade of cellular and molecular events distal to a nerve injury that releases a large spectrum of molecules originating from the disintegration of axons and their myelin sheaths and the extracellular matrix of the endoneurium [6,7,8,9]. The peripheral branches of afferent axons and their terminals contain abundant mitochondria that disintegrate in locations distal to a nerve injury [10,11,12], releasing mitochondrial proteins and DNA (mtDNA). These factors are collectively termed mitochondrial damage-associated molecular patterns (mtDAMPs) [13,14]. mtDNA acts as a ligand for the toll-like receptor 9 (TLR9) that is one of key participants in innate immune responses [15].

One of the early consequences of the neuronal innate immune reaction to axotomy results in the production of a specific collection of immune mediators, such as cytokines and chemokines that are different from injury-induced axon degeneration [16]. The bodies of primary sensory neurons react to peripheral nerve injury together with their satellite glial cells (SGCs) and upregulate cytokines or chemokines. We demonstrated that a unilateral nerve injury not only affects associated DRG but also affects DRG non-associated with the injured nerve, implying a systemic response to WD. We also found that upregulated Interleukin 6 (IL-6) and its transforming factor STAT3 are involved in activating a pro-regenerative state in DRG neurons non-associated with the injured sciatic nerve [17,18,19].

We hypothesize that mtDNAs released from injured axons could likely induce neuroinflammatory reactions in DRG non-associated with the injured nerve, and therefore the levels of the corresponding receptor should be regulated in the DRG. Therefore, we set out to investigate mRNA and protein levels of TLR9 in DRG associated and non-associated with partial or complete sciatic nerve injury. In addition, we investigated a possible role for TLR9 in the neuronal innate immune reaction, which is linked to the induction of a pro-regenerative state in DRG neurons non-associated with nerve lesion. For this purpose, we analyzed NFκB that is an integral component of signal transduction of TLR9 and activation of STAT3 as a marker of neuronal regeneration program.

## 2. Results

### 2.1. Increased Levels of TLR9 Protein and mRNA in DRG Following Sciatic Nerve Lesion

#### 2.1.1. Immunofluorescence Staining

**Figure 1:**
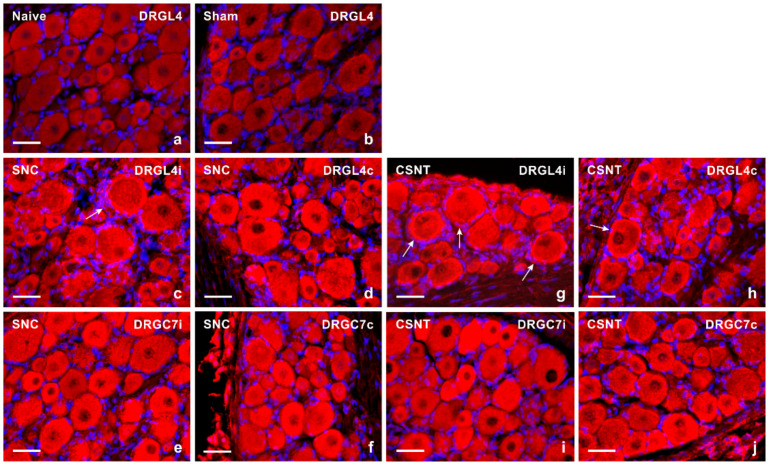
Representative sections of DRG from naive rat (**a**) and rats with sterile sham- (**b**), SNC- (**c**–**f**) or CSNT- (**g**–**j**) interventions for 7 days. The DRG sections of lumbar (DRG-L4) and cervical (DRG-C7) segments of both ipsilateral (**i**) and contralateral (**c**) sides were immunostained for TLR9 using a rabbit polyclonal antibody and donkey TRITC-conjugated anti-rabbit secondary antibody under the same conditions. Besides neuronal bodies, increased TLR9 immunofluorescence was found in satellite glial cells enveloping the neuronal bodies of ipsilateral DRG-L4 after SNC (arrow in Figure 1c) and bilaterally in DRG-L4 after CSNT (arrows in Figure 1g,h). Scale bars = 40 µm.

DRG sections incubated under the same conditions showed very low intensity of TLR9 immunofluorescence in the neuronal cytoplasm of small- and medium-sized neurons in the lumbar and cervical DRG removed from naive and sham-operated rats (Figure 1a,b). The proportion of TLR9 immunopositive neurons was 32.5 ± 6.6% in the lumbar and 23.1 ± 6.2% in the cervical DRG of naive rats. Sham operation induced a slightly increased proportion of immunopositive small- and medium-sized neurons to 35.4 ± 5.6% and 24.8 ± 5.7% in the lumbar and cervical DRG, respectively. Sciatic nerve lesion (both SNC and CSNT) resulted in a significantly increased intensity of TLR9 immunofluorescence in DRG neurons of all size types in both the lumbar and cervical DRG (Figure 1c–j, Table 1). Apart from neurons, increased immunostaining was also found in the satellite glial-like cells enveloping neuronal bodies of ipsilateral DRG-L4 after SNC (Figure 1c) and bilaterally in DRG-L4 after CSNT (Figure 1g,h).

#### 2.1.2. Double Immunostaining

**Figure 2:**
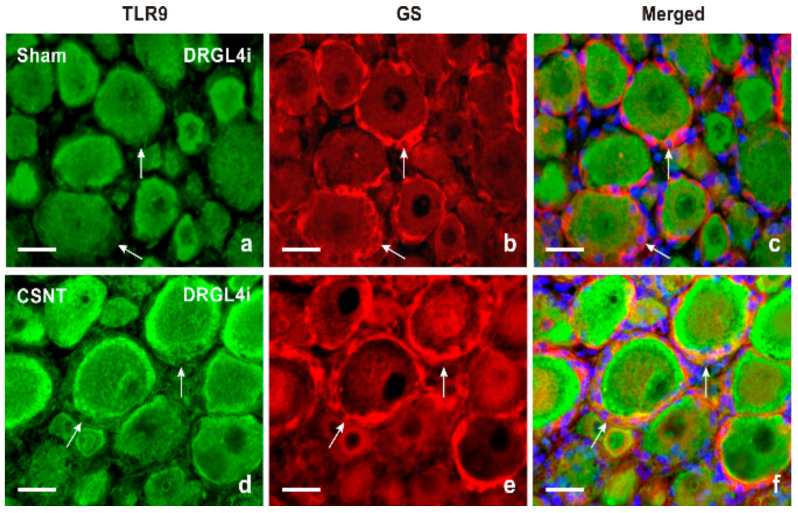
Double immunofluorescence staining for TLR9 and GS as a marker of satellite glial cells (SGCs). Sections of lumbar DRG ipsilateral (DRGL4i) to sham operation (Sham) and CSNT were immunostained with a mixture of rabbit polyclonal anti-TLR9 (**a**,**d**) and mouse monoclonal anti-GS (**b**,**e**) antibodies. Immunostaining was visualized with TRITC-conjugated donkey anti-rabbit and FITC-conjugated donkey anti-mouse secondary antibodies. Cell nuclei were detected with Hoechst 33342. Merged pictures (**c**,**f**) show no TLR9 immunostaining in SGCs of DRG from sham-operated rat (arrows in Figure 2c), while SGCs enveloping large-sized neuronal bodies in DRG from CSNT-operated rat displayed clear TLR9 immunofluorescence (arrows in Figure 2f). Scale bars = 20 µm.

**Figure 3:**
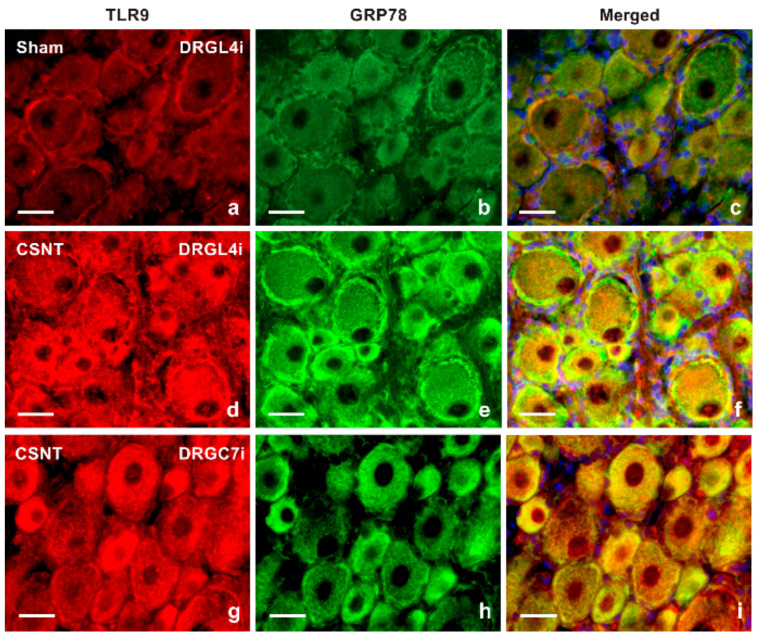
Double immunofluorescence staining for TLR9 and GRP78 as a marker of the endoplasmic reticulum (ER). Sections of lumbar DRG ipsilateral (DRGL4i) to sham operation (Sham) as well as DRGL4i and cervical DRG ipsilateral (DRGC7i) to CSNT were immunostained with mouse monoclonal anti-TLR9 (**a**,**d**,**g**) and rabbit polyclonal anti-GRP78 (**b**,**e**,**h**) antibodies and were visualized using donkey TRITC-conjugated anti-mouse and FITC-conjugated anti-rabbit secondary antibodies, respectively. Cell nuclei were stained with Hoechst 33342. Merged pictures (**c**,**f**,**i**) detected no or very weak TLR9 immunostaining in the ER of DRG neurons from the sham-operated animal (**c**), while most increased TLR9 immunofluorescence was co-localized with GRP89 in the ER of DRGL4i (**f**) and DRGC7i (**i**) from CSNT-operated rats. Scale bars = 20 µm.

**Figure 4:**
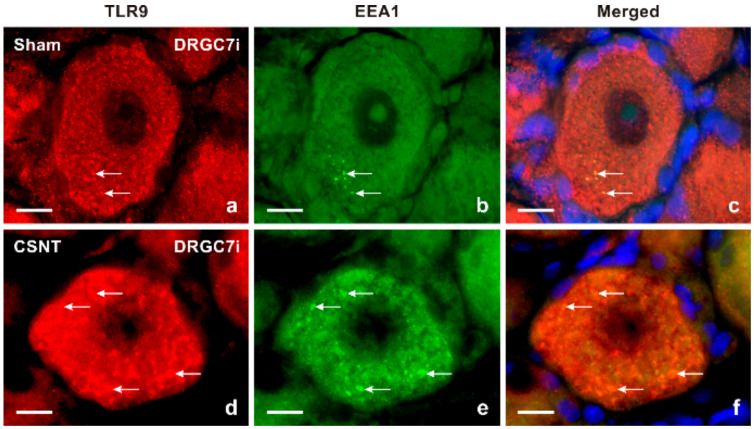
Double immunofluorescence staining for TLR9 and early endosomal antigen 1 (EEA1) as a marker of early endosomes. Sections of cervical DRG ipsilateral (DRGC7i) to sham operation (**a**–**c**) and CSNT, (**d**–**f**) were immunostained with rabbit polyclonal anti-TLR9 (**a**,**d**) and mouse monoclonal anti-EEA1 (**b**,**e**) antibodies. TLR9 and EEA1 were visualized with TRITC-conjugated donkey anti-rabbit and FITC-conjugated donkey anti-mouse secondary antibodies, respectively. Cell nuclei were detected with Hoechst 33342. A limited number of punctate TLR9 and EEA1 co-immunostaining demonstrated TLR9 protein in early endosomes of cervical DRG neurons of sham-operated rat (arrows in Figure 4a–c). More such punctate double immunostaining was found in the neurons of DRGC7i from CSNT-operated rat (arrows in Figure 4d–f). Scale bars = 10 µm.

Results of immunostaining with antibodies against both TLR9 and glutamine synthetase (GS) as a marker of activated SGCs confirmed a slight increase in TLR9 immunofluorescence in activated SGCs surrounding mainly large neuronal bodies of lumbar DRG following sciatic nerve lesion when compared with sham-operated control (Figure 2a–f). The granular pattern of increased TLR9 immunofluorescence in the neuronal bodies of both cervical and lumbar DRG after SNC and CSNT was due to TLR9 localization in the endoplasmic reticulum, as shown by double immunostaining with ER-located GRP78 (Figure 3). Alongside the granular distribution, some large neuronal bodies of DRG from sham-, SNC- and CSNT-operated rats also showed a vesicular pattern of TLR9 immunostaining corresponding with its location in early endosomes, as shown by colocalization of TLR9 and EEA1 (Figure 4). Nevertheless, double immunostaining of TLR9 and Rab7 did not demonstrate localization of TLR9 protein in late endosomes (data not shown). The vesicular localization of TLR9 immunostaining was not observed in naive DRG.

#### 2.1.3. Western Blot Analysis

**Figure 5:**
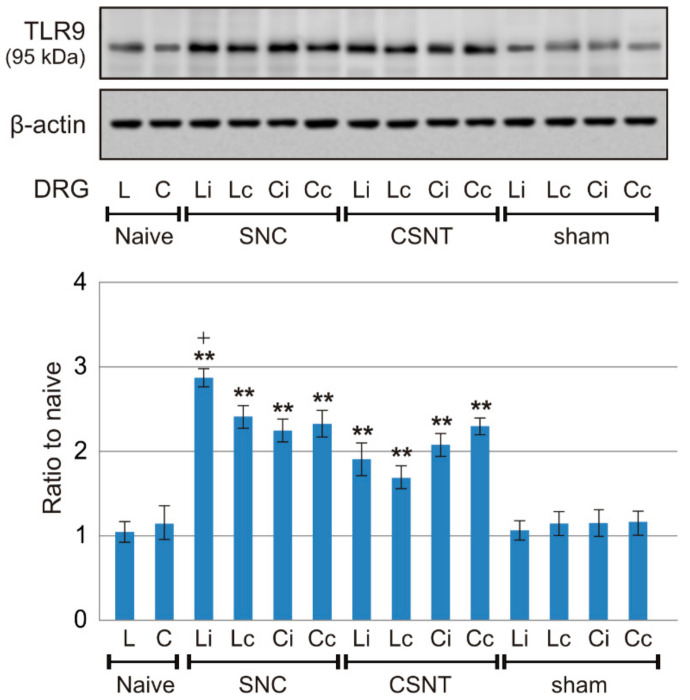
Results of Western blot analysis of TLR9 protein levels. TLR9 protein levels were analyzed in intact lumbar (L) and cervical (C) DRG of naive rats as well as in ipsi- and contralateral lumbar (Li, Lc) and cervical (Ci, Cc) DRG from SNC-, CSNT- and sham-operated rats (*n* = 6 for each group). Upper panels illustrate representative Western blot bands of TLR9 and actin used to confirm equal loading of proteins. The lower panel shows densitometry of TLR9 protein bands (mean density ± SD) after normalization to actin; the intensities of the TLR9 bands from naive DRG were taken as 1. ** Significant difference (*p* < 0.01) compared with naive or sham controls, + significant difference (*p* < 0.05) compared ipsi- and contralateral DRG in a Kruskal–Wallis ANOVA test.

To verify the immunohistochemical results showing a bilateral increase in TLR9 protein levels in DRG of both lumbar and cervical segments after unilateral sciatic nerve injury, protein levels were analyzed in a Western blot. TLR9 protein levels were significantly increased in both lumbar and cervical DRG of both sides after unilateral SNC and CSNT, compared with controls from naive or sham-operated rats. Lumbar DRG ipsilateral to SNC showed a significantly higher TLR9 protein level than those contralateral to SNC, while the TLR9 protein accumulation was similar on both sides in cervical DRG. Although not statistically significant, a similar tendency of TLR9 protein accumulation was found in lumbar DRG following CSNT (Figure 5).

#### 2.1.4. Real-Time RT-PCR 

**Figure 6:**
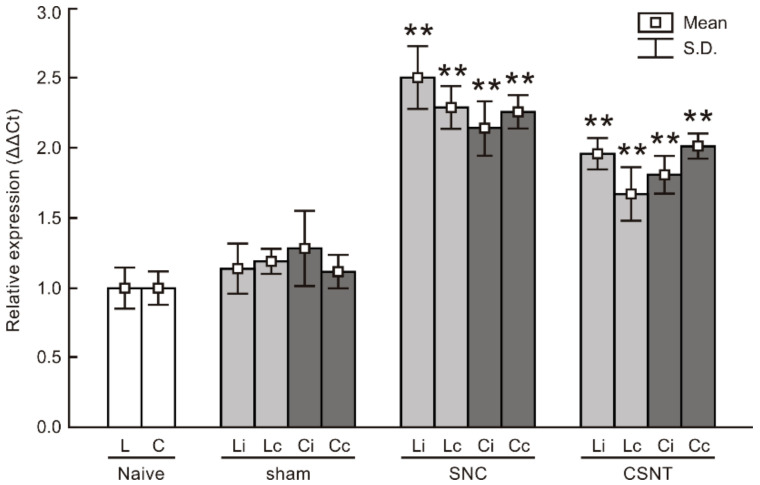
Results of real-time PCR (RT-PCR) of relative TLR9 mRNA levels in DRG of lumbar (L) and cervical (C) spinal segments from ipsilateral (i) and contralateral (c) sides of naive as well as sham-, SNC- and CSNT-operated rats for 7 days (*n* = 6 for each group). Relative expressions were determined using actin as the housekeeping gene and normalized to naive controls. ** Significant difference (*p* < 0.01) compared with naive or sham-operated rats in a Kruskal–Wallis ANOVA test.

RT-PCR was used to determine the levels of TLR9 mRNA in cervical and lumbar DRG 7 days after unilateral sciatic nerve injury by SNC or CSNT. Samples of cervical and lumbar DRG from sham-operated animals displayed no significant changes in TLR9 mRNA levels compared with naive controls. Levels of TLR9 mRNA were significantly increased bilaterally in both cervical and lumbar DRG one week after the sciatic nerve lesion by SNC or CSNT compared with naive or sham-operated controls. We found no statistically significant difference between ipsilateral and contralateral cervical or lumbar DRG (Figure 6).

### 2.2. TLR9 and Neuronal Innate Immune Reactions Associated with Pro-Regenerative State of DRG Neurons

**Figure 7:**
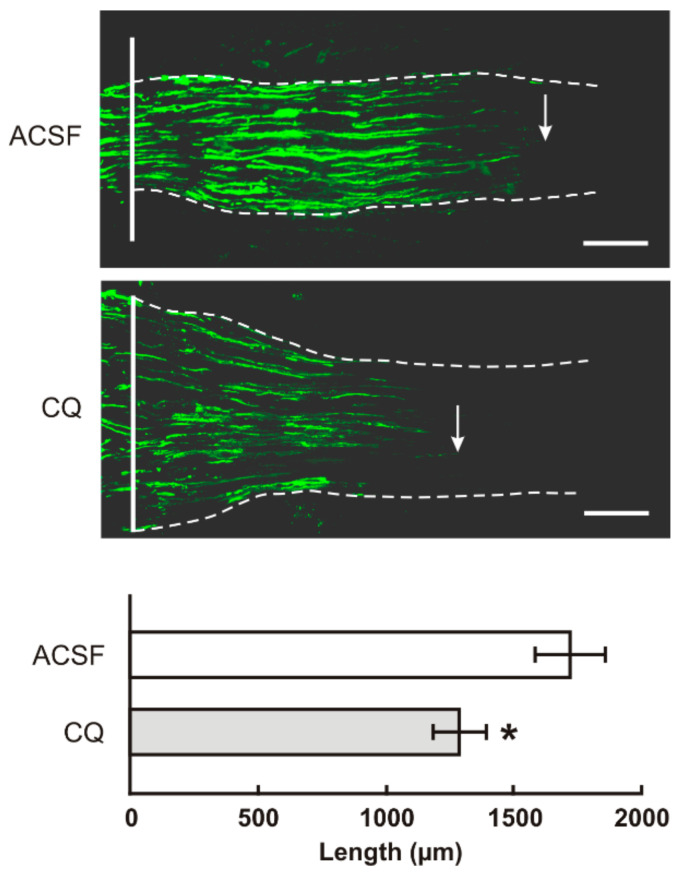
The top panels illustrate representative longitudinal sections through ulnar nerves distal to the crush site (solid line) showing SCG10 immunopositive regenerated axons. Arrows indicate the tip of the longest SCG10 immunopositive axons regenerated 1 day after the ulnar nerve crush in rats with prior CSNT for 7 days and intrathecal application of artificial cerebrospinal fluid (ACSF) or chloroquine (CQ). Scale bars = 280 µm. The bottom panel illustrates mean length of regenerated SCG10 immunopositive axons ± SD in the ulnar nerve 1 day after crush and 7 days from intrathecal application of artificial cerebrospinal fluid (ACSF) or CQ; *n* = 6 for each group. * Significant difference (*p* < 0.05) compared with ACSF in a Kruskal–Wallis ANOVA test.

We observe a bilateral increase of TLR9 protein and mRNA following unilateral sciatic nerve lesion similar to IL6 and activation of STAT3 [17,18,19]. We therefore tested whether increased TLR9 is also associated with the pro-regenerative state of cervical DRG. For this we used an in vivo model of sciatic nerve injury followed by measuring the length of the regenerated afferent axons distal to the ulnar nerve crush [19]. We found that the axon regeneration index expressed as SCG10 positive axon extension from the point of ulnar nerve crush was significantly reduced in CSNT-operated rats treated with chloroquine (CQ) compared with intrathecal application of artificial cerebrospinal fluid (ACSF) (Figure 7).

**Figure 8:**
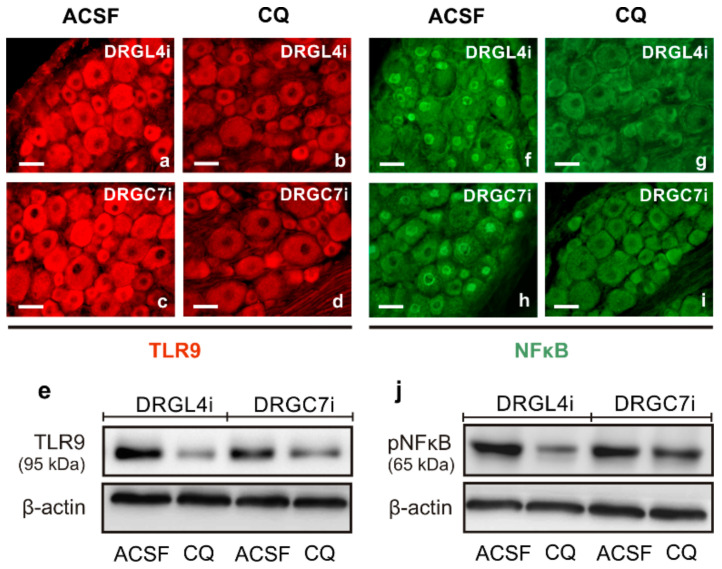
Immunostaining for TLR9 (**a**–**d**) and pNFκB (**f**–**i**) in the lumbar (DRGL4i) and cervical DRG (DRGC7i) ipsilateral to the crushed ulnar nerve in rat with prior CSNT for 7 days and intrathecal application of artificial cerebrospinal fluid (ACSF) or chloroquine (CQ). Sections were immunostained with rabbit polyclonal anti-TLR9 or rabbit monoclonal anti-pNFκB(p65) antibodies and were visualized using TRITC- or FITC-conjugated donkey anti-rabbit secondary antibodies, respectively. Immunostaining demonstrated intense immunofluorescence of both TLR9 and pNFκB in the DRG neurons of rat treated with ACSF (**a**,**c**,**f**,**h**), but intrathecal application of CQ reduced the intensity of both TLR9 and pNFκB immunofluorescence (**b**,**d**,**g**,**i**). All sections were processed under the same conditions. Scale bars = 40 µm. Decreased protein levels of both TLR9 and pNFκB in the DRGL4i and DRGC7i after CQ application was verified by Western blotting (**e**,**j**).

**Figure 9:**
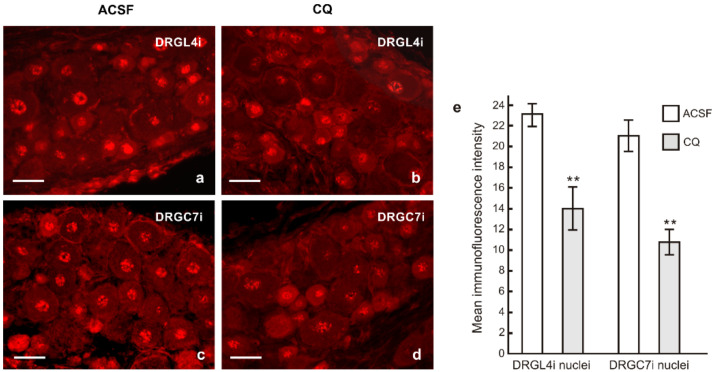
Representative sections of the lumbar (DRGL4i) and cervical DRG (DRGC7i) ipsilateral to the crushed ulnar nerve in rat with prior CSNT for 7 days and intrathecal application of artificial cerebrospinal fluid (ACSF, (**a**,**c**)) or chloroquine (CQ, (**b**,**d**)). Sections were immunostained with rabbit polyclonal anti-STAT3(Y705) and TRITC-conjugated donkey anti-rabbit antibodies under the same conditions to demonstrate nuclear translocation of activated STAT3. Scale bars = 40 µm. The graph (**e**) shows decreased intensity of STAT3 immunofluorescence in the DRG neuronal nuclei after CQ application. ** Significant difference (*p* < 0.01) compared with ACSF in a Kruskal–Wallis ANOVA test.

Given the shorter lengths of regenerating axons following intrathecal CQ application, we also tested changes in pNFκB and nuclear translocation of activated STAT3 in the cervical DRG neurons in the same experimental model. Immunostaining of TLR9 and pNFκB under the same conditions showed decreased intensities of both TLR9 and pNFκB immunofluorescence in the lumbar as well as cervical DRG neurons following intrathecal CQ application compared with ACSF controls. These results were verified by Western blot analysis (Figure 8). Moreover, the pro-regenerative state of cervical DRG neurons induced by sciatic nerve lesion was associated with nuclear translocation of STAT3 [17]. Intrathecal CQ application not only reduced TLR9 and pNFκB levels but also reduced nuclear translocation of STAT3 (Y705) in both the lumbar and cervical DRG after CSNT and ulnar nerve crush (Figure 9).

## 3. Discussion

Wallerian degeneration distal to nerve injury releases a large spectrum of DAMPs produced by cleavage of the endoneurial extracellular matrix and disintegration of axonal plasma membrane and mitochondria [8,20]. Motor and afferent axons in peripheral nerves contain large numbers of mitochondria, which then become a potent source of mtDNA following their disintegration during WD. The mtDNAs produced by WD, as in any other tissue injury, can be released into the bloodstream and induce inflammatory reactions in tissue remote from the site of primary trauma [21]. mtDNA get into the bloodstream because the blood–nerve barrier is interrupted in distal segments of the injured nerve [22]. Moreover, DRG are free of the blood–nerve barrier [23,24] and so presumably mtDNA from disintegrated axonal mitochondria enter the bloodstream, are transported by circulating blood, and diffuse into remote cervical DRG. Thus, while mtDAMPs can penetrate DRG, the question arises as to whether the primary sensory neurons express TLR9, the receptor for mtDNA [25,26]. However, current information about TLR9 expression in primary sensory neurons following nerve injury is very limited and controversial. When isolated from DRG of mouse embryos and cultivated in vitro, primary sensory neurons did not express TLR9 mRNA or protein [27]. In contrast, Qi and coworkers [28] demonstrated TLR9 in primary culture of mouse DRG neurons isolated from E13 embryos and increased TLR9 level following treatment with a corresponding ligand.

Our results of immunofluorescence detection and Western blot analysis show increased levels of TLR9 protein bilaterally in DRG neurons both associated and non-associated with unilateral sciatic nerve lesion caused by SNC and CSNT. This bilateral increase in TLR9 synthesis in lumbar and cervical DRG following unilateral sciatic nerve lesions was confirmed by RT-PCR.

### 3.1. Intracellular Localization of TLR9 in DRG Neurons

In contrast to membrane-bound TLRs (TLR2, TLR4), it is generally accepted that TLR9 is initially located in the endoplasmic reticulum and is translocated to endosomes upon stimulation by CpG DNA [29]. Double immunostaining with TLR9 and GRP78 antibodies showed that the increased TLR9 immunofluorescence was predominantly in the endoplasmic reticulum of DRG neurons of SNC- or CSNT-operated rats. GRP78/BiP is a major protein chaperone critical for protein quality control in the endoplasmic reticulum [30], and GRP78 immunostaining is used as a reliable marker of the endoplasmic reticulum in the DRG neurons [31].

TLR9, while initially located in the endoplasmic reticulum, is translocated to TLR9 ligand-containing endosomes, which also accumulate MyD88 [29,32]. Double immunostaining revealed colocalization of TLR9 and EEA1 immunofluorescence, indicating that a portion of TLR9 is translocated into early endosomes of DRG neurons after sham operation and sciatic nerve lesion, which corresponds to the results obtained in immune cells [29]. However, most TLR9 immunostaining remained in the endoplasmic reticulum of DRG neurons.

### 3.2. Neuronal Innate Immune Reaction and the Pro-Regenerative State of DRG Neurons

But what is the role of increased TLR9 level in DRG neurons associated and non-associated with sciatic nerve lesions? It has been demonstrated recently that primary sensory neurons have an intrinsic innate immune capacity in response to traumatic axonal injuries associated with upregulation of a specific collection of cytokines and chemokines [16]. Further, it is generally accepted that TLR9 activation by mtDNA can regulate innate immune responses [26]. The results we show here of bilateral upregulation of TLR9 are similar to our previous observations that unilateral sciatic nerve injury induced bilateral upregulation of the cytokine IL6 and its signaling pathway, including activation of STAT3, as well as the chemokine CXCL12 and its receptor CXCR4, not only in lumbar but also in cervical DRG neurons [17,33,34]. These upregulations correlate well with our current results on activated NFκB, the key transcription factor in the regulation of cytokines and chemokines acting downstream of TLR9 [25]. The results here, taken together with our previous observations, suggest that mtDNA released during WD following unilateral sciatic nerve lesion is able to activate the TLR9 signaling cascade, including NFκB in DRG neurons, to regulate neuronal cytokines and chemokines.

It was demonstrated that IL6-activated STAT3 initiates the neuronal regeneration program [5,35,36,37]. We also found that upregulation of IL6 in cervical DRG induced activation of STAT3, indicating a pro-regenerative state of the primary sensory neurons non-associated with unilateral sciatic nerve lesion. This was detected by measuring the length of SCG10 immunopositive regenerated axons distal to the ulnar nerve crush after a prior sciatic nerve lesion [17,18,19]. The same rat model, when used here with intrathecal CQ application, showed that shorter SCG10 immunopositive axons regenerated distal to the ulnar nerve crush compared with control rats. This inhibitory effect of CQ on ligand binding with the activated form of TLR9 [38,39] was accompanied by a decrease in TLR9 and pNFκB protein levels, as well as by a reduced intensity of nuclear translocated STAT3 in cervical DRG neurons. We cannot assess the direct effect of CQ to STAT3 or NFkB in our in vivo experiments. However, it was demonstrated that CQ treatment may reduce STAT3 in cancer stem cells cultivated in vitro [40]. Although cancer and cancer stem cells express a high level of TLR9 [41,42], whether a decrease of STAT3 can occur via reduction of TLR9-mediated signaling by CQ application was not investigated in this paper. In contrast, CQ treatment increased activation of NFκB in squamous cell carcinoma and melanoma tumor cells through the accumulation of autophagosomes [43]. On the contrary, CQ inhibited MGC803 gastric cancer cell migration via the TLR9/NFκB signaling pathway [44], supporting our results of decreased NFκB in the DRG neurons after intrathecal application of CQ.

TLR9 was also detected in the growth cones, indicating that these critical structures of regenerated axons can also react to mtDNA distal to nerve injury [45]. A role for TLR9 in axonal regeneration has been suggested [46], but direct evidence for this TLR9 function is not yet available. Intrathecal application of CQ can affect the growth of regenerated axons either via local inhibition of ligand binding with the activated form of TLR9 on growth cones or directly by attenuating the TLR9-mediated neuronal innate immune reaction and thereby reducing regeneration capacity.

In conclusion, our results indicate that a unilateral sciatic nerve lesion activates the neuronal innate immune reaction via TLR9 and NFκB not only in DRG associated with the injured nerve but also in remote DRG. The neuronal innate immune reaction can induce a regenerative state in DRG neurons associated with the injury and a pro-regenerative state in neurons non-associated with the injured nerve. Nevertheless, what has not so far been elucidated is the purpose of conditioning primary sensory neurons of remote DRG in reaction to a unilateral sciatic nerve lesion.

## 4. Materials and Methods

### 4.1. Animals and Surgical Treatment

The investigation was performed in 96 adult male rats (Wistar, 250–280 g, Anlab, Brno, Czech Republic) housed in 12 h light/dark cycles at a temperature of 22–24 °C under specific pathogen-free conditions in the animal housing facility of Masaryk University. Sterilized standard rodent food and water were available ad libitum. All experimental procedures were carried out under sterile conditions by the same person according to protocols approved by the Animal Investigation Committee of the Faculty of Medicine, Brno, Czech Republic. All surgical procedures were performed under anesthesia using a mixture of ketamine (40 mg/mL) and xylazine (4 mg/mL) administered intraperitoneally (0.2 mL/100 g body weight).

The right sciatic nerve was exposed in the mid-thigh, ligated using 2 ligatures and cut with a pair of sharp scissors (complete sciatic nerve transection, CSNT, *n* = 18). The proximal nerve stump was fixed in muscles to shield the distal stump from reinnervation. A 2 mm long silicone tube of 1 mm internal diameter was slit longitudinally and placed around the right sciatic nerve to reduce nerve diameter (sciatic nerve constriction, SNC, *n* = 18) [47]. The tube was tied with a sterile thread to prevent opening. The muscles and skin were closed with 5/0 sutures. The right sciatic nerve of sham-operated rats (*n* = 18) was carefully exposed without any lesion. All operated rats (CSNT, SNC, sham) were allowed to survive for 7 days, a period of time where WD has fully developed distal to the nerve injury and when peak expression of regeneration-associated proteins in DRG neurons has occurred [19]. Eighteen other rats without any surgical treatment were used as naive control. DRG from rats belonging to naive, sham-, CSNT- and SNC-groups were used for simple or double immunohistochemical staining as well as Western blot and RT-PCR analysis.

### 4.2. Tissue Processing and Immunofluorescence Staining

Naive, sham-, SNC- and CSNT-operated rats (*n* = 6 for each group) were deeply anesthetized with a lethal dose of sodium pentobarbital (70 mg/kg body weight, i.p.) and perfused transcardially with 400 mL phosphate-buffered saline (PBS, pH 7.4) followed by 400 mL of Zamboni’s fixative [48]. We demonstrated the phenomenon of a pro-regenerative state in the DRG neurons of cervical (C6-C8) spinal segments that are non-associated with unilateral injury of the sciatic nerve [18,19]. Therefore, we used the DRG of the same cervical segments for the current analysis. Rat lumbar DRG of L4-L5 segments contain nearly 98–99% of the primary sensory neurons that contribute to the rat sciatic nerve [49]. The L4-L5 DRG associated with the sciatic nerve were used for comparison with remote DRG (C6-C8). The L4-L5 and C6-C8 DRG from both sides were detected inside their intervertebral foramina following total laminectomy and foraminotomy. The DRG were removed, immersed separately in Zamboni’s fixative at 4 °C overnight and then divided into samples of ipsilateral (L-DRGi) and contralateral (L-DRGc) lumbar DRG as well as ipsilateral (C-DRGi) and contralateral (C-DRGc) cervical DRG separately for each group of rats (naive, sham-, SNC- and CSNT-operated). The DRG samples were washed in 20% phosphate-buffered sucrose for 12 h, blocked in Tissue-Tek^®^ OCT compound (Miles, Elkhart, IN, USA), and serial longitudinal cryostat sections (12 µm) were prepared. The sections were then mounted on chrome-alum covered slides and processed for indirect immunofluorescence staining.

Briefly, DRG sections of lumbar and cervical segments of naive, sham-, SNC- and CSNT-operated rats were immunostained simultaneously under the same conditions. Sections were washed with PBS containing 0.05% Tween 20 (PBS-T) and 1% bovine serum albumin (BSA) for 10 min and were treated with 5% normal donkey serum in PBS-T for 30 min. They were then incubated with 25 μL of a polyclonal rabbit antibody against TLR9 (1:500; AP05241PU-N, Acris, Herford, Germany) in a humid chamber at room temperature (21 to 23 °C) for 12 h. The immunostaining was visualized using TRITC-conjugated and affinity-purified donkey anti-rabbit secondary antibody (1:100; AP182R, EMD Millipore, Tamecula, CA, USA) for 90 min at room temperature. Control sections were incubated without primary antibodies, with preabsorbed antibody (20 µg/mL, TLR9 blocking peptide, MBS-152758, MyBioSource, San Diego, CA, USA) or by substituting the primary antibodies with the donkey IgG isotype. The sections were stained with Hoechst 33,342 to detect cell nuclei, mounted in aqueous mounting medium (Vectashield; Vector Laboratories, Burlingame, CA, USA) and analyzed using an epifluorescence microscope (Nikon Eclipse) equipped with a Nikon DS-Ri1 camera (Nikon, Prague, Czech Republic) and a stabilized power supply for the lamp housing. The TLR9 immunofluorescence intensities were measured using a NIS-Elements image analysis system (Nikon, Prague, Czech Republic) according to our previously published protocol [19]. Briefly, TLR9 immunofluorescence intensities were measured in digital pictures of the RGB mode captured under the same magnification (Plan Fluor objective 40 × 0.75; Nikon) with a Nikon DS-Ri1 camera. After subtraction of background, the DRG neurons were detected by the interactive thresholding technique (HSI: hue, saturation and intensity) and transformed to binary mode. The binary foreground was monitored at every step of thresholding and manually edited if needed. The original RGB true color images were converted to gray and overlaid with the binary map. At least 100 neuronal profiles containing nuclei were measured for each animal group. The DRG neurons were categorized as small (<25 μm), medium (25–40 μm) or large (>40 μm).

Measurement of immunofluorescence intensities on digitized images from an epifluorescence microscope is a simple and rapid method providing reliable information on the cellular distribution and comparison of protein levels between control and experimental tissue samples, for example in DRG neurons [19,33,34].

### 4.3. Double Immunostaining

To detect cellular and intracellular TLR9 localization, we performed double immunostaining of TLR9 with antibodies against glutamine synthetase (GS) as a marker of activated SGCs [50] and against early endosomal antigen-1 (EEA1) or Rab7 to detect early or late endosomes, respectively [51,52]. Further we used a polyclonal antibody against glucose-regulated protein 78 (GRP78) in double immunostaining to monitor TLR9 localization to the endoplasmic reticulum [53].

Briefly, one portion of the DRG sections was incubated with a mixture (1:1) of rabbit polyclonal anti-TLR9 and mouse monoclonal anti-GS (1:100; LS-B2579/67910 LS Bio, Seattle, WA, USA) or anti-EEA1 (1:50; sc-53939, Santa Cruz, CA, USA) antibodies. Another portion was incubated with mouse monoclonal anti-TLR9 (1:100; sc-52966, Santa Cruz Biotechnology, Heidelberg, Germany) and one of either rabbit monoclonal anti–Rab7 (1:100; #9367, Cell Signaling Technology, The Netherlands) or rabbit polyclonal anti-GRP78 (1:500; ab53068, Abcam, Cambridge, UK) antibodies. A mixture (1:1) of affinity purified TRITC-conjugated donkey anti-rabbit and FITC-conjugated donkey anti-mouse secondary antibodies (1:100; AP182R and AP192F, EMD Millipore, Tamecula, CA, USA) was applied at room temperature for 90 min. Control sections were incubated without the primary antibodies, with preabsorbed polyclonal TLR9 antibody (20 µg/mL, TLR9 blocking peptide, MBS-152758, MyBioSource, San Diego, CA, USA), preabsorbed monoclonal antibody using recombinant human TLR9 protein (10 µg/mL, TLR9-48H, Creative-Biomart, Shirley, NY, USA) or with a reverse combination of primary and secondary antibodies. The control sections displayed no immunostaining. The sections were stained with Hoechst 33,342 to detect cell nuclei, mounted in aqueous mounting medium (Vectashield; Vector Laboratories, Burlingame, CA, USA) and analyzed using appropriate epifluorescence optics with fluorescent filter cubes for TRITC, FITC and UV (TRITC: excitation filter 554 nm, barrier filter 609 nm; FITC: excitation filter 466 nm, barrier filter 525 nm; UV: excitation filter 378 nm, barrier filter 447 nm) on a Nikon Eclipse microscope equipped with a Nikon DS-Ri1 camera (Nikon, Prague, Czech Republic).

### 4.4. Western Blot Analysis

Naive, sham-, SNC- and CSNT-operated rats (*n* = 6 for each experimental group) were deeply anesthetized with a lethal dose of sodium pentobarbital (70 mg/kg body weight, i.p.). DRG of both sides were then detected following total laminectomy and foraminotomy, removed under aseptic conditions, washed in protease and phosphatase inhibitor cocktails (Roche, Germany), flash frozen in liquid nitrogen and stored at −80 °C until further analysis. The DRG of lumbar (L4-L5) and cervical (C6-C8) segments were separated into samples of ipsilateral (L-DRGi) and contralateral (L-DRGc) lumbar DRG as well as ipsilateral (C-DRGi) and contralateral (C-DRGc) cervical DRG for each group of rats. For triplicate Western blotting analysis of TLR9, samples of DRG were collected from two rats in each sample group. The DRG samples were homogenized in TRIS-buffered saline (pH 7.2) containing 0.1% Triton X-100 and protease and phosphatase inhibitors and centrifuged at 10,000× *g* for 5 min at 4 °C. The total protein concentration was measured in the tissue supernatant (Nanodrop ND-1000; Thermo Fisher Scientific) and normalized to the same levels. Proteins were separated by SDS-polyacrylamide gel electrophoresis and transferred to nitrocellulose membranes by electroblotting (BioRad, Hercules, CA, USA). Blots were blocked using 1% BSA in PBS-T (pH 7.4) for 1 h and incubated with rabbit polyclonal anti-TLR9 (1:1000; AP05241PU-N, Acris, Germany) or rabbit monoclonal anti-pNFκB(p65) (1:1000, #3033, Cell Signaling Technology, Danvers, MA, USA) antibodies overnight. After washing in PBS-T, blots were incubated with peroxidase-conjugated anti-rabbit IgG (1:1000; A2074, Sigma-Aldrich, Saint Louis, MO, USA) at room temperature for 1 h. Equal loading of proteins was confirmed by actin staining. Protein bands were visualized using the ECL detection kit (Cytiva, Marlborough, MA, USA) on a chemiluminometer reader (LAS-3000; Fuji, Japan) and analyzed using image densitometry software. The levels of proteins were normalized to β-actin and to the value of naive lumbar and cervical DRG, which were arbitrarily set to one.

### 4.5. Real Time RT-PCR

The expression of TLR9 mRNAs in DRG was analyzed by real time PCR (RT-PCR). Whole DRG were removed under aseptic conditions from lumbar (L4-L5) and cervical (C6-C8) segments of both sides from naive, sham-, SNC-, CSNT-operated rats (*n* = 6 for each group). The samples were collected from ipsilateral and contralateral lumbar DRG (L-DRGi, L-DRGc) as well as ipsilateral and contralateral cervical DRG (C-DRGi, C-DRGc) for each group of rats and stored in RNA later (Thermo Fisher Scientific, Waltham, MA, USA) at 4 °C. First-strand cDNA synthesis was performed using TaqMan^®^ High-Capacity RNA-to-cDNA Kit (Thermo Fisher Scientific, Waltham, MA, USA), and their quality and concentrations were evaluated by optical density using NanoDrop (Labtech France, Palaiseau, France). PCR amplification, in triplicate for each sample, was performed using ABI Prism 7300, TaqMan^®^ Gene Expression Master Mix and TaqMan^®^ Gene Expression Assay Probes FAM™ (Thermo Fisher Scientific, Waltham, MA, USA) for the target gene TLR9 (assay ID- Rn01640054_m1). Determinations were made with reference to a reporter gene encoding Rat Actin (actin, beta-Rn00667869_m1) Endogenous Control (VIC^®^). The polymerase activation step at 95 °C for 15 min was followed by 40 cycles of 15 s at 95 °C and 60 s at 60 °C. The validity of the results was checked by running appropriate negative controls (replacing cDNA with water for PCR amplification; omitting reverse transcriptase for cDNA synthesis). Specific mRNA levels were calculated after normalizing to actin mRNA in each sample. Relative expression was determined using the Comparative Ct Model (ΔΔCt) with actin as the housekeeping gene. Data were presented as relative mRNA units compared with control values (expressed as fold multiples of the appropriate sham control).

### 4.6. Intrathecal Administration of Chloroquine, In Vivo Axon Regeneration Assay and Evaluation of Transforming Factors

To test in vivo the role of TLR9 in sciatic nerve injury triggering the pro-regenerative capacity of cervical DRG neurons, we used the rat ulnar nerve crush model with prior CSNT for 7 days [19]. The right ulnar nerves of 12 rats surviving 7 days with CSNT were exposed for a short segment and crushed using a clamp with a defined force of 1.9 N for 1 min twice [54] under a stereoscopic microscope. The distal margin of the crush injury was indicated with Indian ink, and the skin wound was closed with 5/0 sutures.

The animals were randomly divided into a control group (*n* = 12) intrathecally injected with artificial cerebrospinal fluid (ACSF) [55] and an experimental group (*n* = 12) who were administered quinoline (CQ) intrathecally. The procedure comprised injecting 10 µL of ACSF or freshly prepared quinoline (InvivoGen, Toulouse, France) in ACSF (5 mg/kg) and a further 10 µL ACSF via a micro syringe into the subarachnoid space of the cisterna magna [17]. Rats with intrathecal administration of ACSF or CQ were left to survive for 1 day.

#### 4.6.1. Axon Regeneration Assay and Immunohistochemical Analysis

Rats with intrathecal administration of ACSF or CQ (*n* = 6 for each group) were deeply anesthetized and perfused transcardially with PBS and Zamboni’s fixative. The lumbar (L4-L5) and cervical DRG (C6-C8) and the ulnar nerve segment distal to the crush point were removed, fixed with Zamboni’s fixative overnight and washed in 10% sucrose in PBS. Serial longitudinal cryostat sections (thickness 10 µm) through ulnar nerve segments were cut and immunostained with rabbit polyclonal antibody against SCG10 (1:1000; LS-C143454/76191, LSBio, Seattle, WA, USA) in a humid chamber at room temperature (21 to 23 °C) for 12 h. The immunoreaction was visualized by treatment with FITC-conjugated and affinity-purified donkey anti-rabbit secondary antibody (1:100; AP192F, EMD Millipore, Tamecula, CA, USA) for 90 min at room temperature. SCG10 fluorescence intensity was analyzed along the length of the nerve distal to the crush site [56]. The length of SCG10 immunopositive axons was measured by a person blind to the experimental conditions in every third section using a NIS-Elements image analysis system (Nikon, Prague, Czech Republic). The results were expressed as mean maximal length ± SD of regenerated axons.

The lumbar and cervical DRG samples of ACSF and CQ treated rats were cut to prepare serial longitudinal cryostat sections (thickness 12 µm). One portion of the sections of ACSF and CQ groups were double immunostained under the same conditions as above using rabbit polyclonal anti-TLR9 (1:500; AP05241PU-N, Acris, Herford, Germany) and rabbit monoclonal anti-pNFκB(p65) (1:100; #3033, Cell Signaling Technology, Danvers, MA, USA) antibodies and the immunoreaction visualized using TRITC-conjugated donkey anti-mouse and FITC-conjugated donkey anti-rabbit secondary antibodies, respectively. Another portion of sections was immunostained with rabbit polyclonal antibody against STAT3(Y705) (1:100; sc-7993, Santa Cruz Biotechnology, Heidelberg, Germany), and the immunohistochemical reaction was visualized using TRITC-conjugated and affinity-purified donkey anti-rabbit secondary antibody (1:100; AP182R, EMD Millipore, Tamecula, CA, USA) for 90 min at room temperature. The sections were stained with Hoechst 33,342 to detect cell nuclei and analyzed using an epifluorescence microscope (Nikon Eclipse, Nikon, Prague, Czech Republic). The STAT3(Y705) immunofluorescence intensities in the nuclei of DRG neurons were measured using the NIS-Elements image analysis system (Nikon, Prague, Czech Republic) according to a protocol we published previously [17]. At least 200 nuclei with visible nucleoli of DRG neurons were measured for each group (ACSF and CQ). The immunofluorescence intensities were expressed as mean intensities ± SD.

#### 4.6.2. Western Blot Analysis

A second group of rats with intrathecal injection of ACSF or CQ (*n* = 6 for each group) were deeply anesthetized with a lethal dose of sodium pentobarbital. The lumbar (L4-L5) and cervical DRG (C6-C8) ipsilateral to CSNT and the ulnar nerve crush were removed under aseptic conditions, washed in protease and phosphatase inhibitor cocktails (both Roche, Mannheim, Germany), flash frozen in liquid nitrogen, and stored at −80 °C until analyzed by Western blot as described above.

### 4.7. Statistical Analyses

All data were expressed as mean ± SD and tested for normal distribution. Statistical differences were tested using a Kruskal–Wallis ANOVA test for comparisons between data of immunofluorescence intensities, Western blot analysis and RT-PCR of naive DRG neurons and DRG neurons of sham-operated rats or rats with SNC and CSNT (* *p* < 0.05; ** *p* < 0.01). The same statistical analysis was used to compare the length of regenerated axons distal to the ulnar nerve crush after intrathecal application of ACSF and CQ. All statistical analyses were performed using STATISTICA 9.0 software (StatSoft, Inc., Tulsa, OK, USA).

## Figures and Tables

**Table 1:** Results from image analysis of TLR9 immunofluorescence intensity in the dorsal root ganglia (DRG) of cervical (C6-C8) and lumbar (L4-L5) spinal segments. The DRG were removed from naive rats (Naive) as well as those subjected to Sham, SNC and CSNT operation (*n* = 6 for each group). The DRG of operated rats were removed from both ipsilateral (Ipsi) and contralateral (Contra) side to unilateral sciatic nerve lesions. The immunofluorescence intensity of TLR9 is expressed as mean ± SD.

**Naive**	**Cervical DRG**		**Lumbar DRG**	
	117.2 ± 20.7		127.9 + 18.2	
	**Ipsi**	**Contra**	**Ipsi**	**Contra**
**Sham**	128.3 ± 16.8	125.3 ± 12.6	139.5 ± 16.2	134.5 ± 17.9
**SNC**	205.8 ± 14.6 **	197.2 ± 20.3 **	209.3 ± 19.3 **	199.7 ± 15.4 **
**CSNT**	206.5 ± 20.2 **	198.6 ± 21.3 **	207.9 ± 20.4 **	190.6 ± 17.4 **

** Significant difference (*p* < 0.01) compared with naive or sham-operated rats in a Kruskal–Wallis ANOVA test.

## Data Availability

The datasets presented in this study are available on request from the corresponding author. Raw data are stored in the authors’ institutional repositories and will be accordingly provided upon request.
